# Measuring Effects of Counseling to Increase Pre-Exposure Prophylaxis Adherence and Partner Support in South Africa Using the Healthy Relationship Assessment Tool

**DOI:** 10.9745/GHSP-D-22-00075

**Published:** 2023-10-30

**Authors:** Seth Zissette, Elizabeth E. Tolley, Andres Martinez, Sarah T. Roberts, Thesla Palanee-Phillips, Elizabeth T. Montgomery

**Affiliations:** a Department of Epidemiology, Rollins School of Public Health, Emory University, Atlanta, GA, USA.; b Behavioral, Epidemiological, & Clinical Sciences, FHI 360, Durham, NC, USA.; c Women's Global Health Imperative, RTI International, Berkeley, CA, USA.; d Faculty of Health Sciences, School of Public Health, Wits RHI, University of the Witwatersrand, Johannesburg, South Africa.; e Department of Epidemiology, School of Public Health, University of Washington, Seattle, USA.

## Abstract

**Introduction:**

In response to the diverse roles and strong influence that male partners may have in women's decisions and ability to use HIV prevention products, we previously developed the counselor-administered Healthy Relationship Assessment Tool (HEART). This tool helps counselors tailor pre-exposure prophylaxis (PrEP) adherence support counseling to women's relationship dynamics with their sexual partners, suggesting modules for counselors to deliver. In this study, we examined the extent to which counselors used the HEART to guide the delivery of intervention modules and whether changes in subsequent HEART scores reflected the counseling module(s) received.

**Methods:**

We conducted this study during a randomized controlled trial of the Community Health clinic model for Agency in Relationships and Safer Microbicide Adherence (CHARISMA) counseling intervention in Johannesburg, South Africa. Trained lay counselors administered the HEART to intervention group participants at enrollment (n=203) and at 3- and 6-month follow-up visits between October 2018 and April 2021. The HEART auto-calculated participants' response scores to recommend relevant counseling modules. We compared the mean scores for the 5 HEART scales across groups receiving different counseling modules. We also assessed changes in scale scores over time by the counseling module received.

**Results:**

Although counselors could override HEART counseling module recommendations based on additional knowledge of participants' cases, they consistently agreed with the HEART recommendations. The HEART also triggered the sorting of women into counseling modules that they were positioned to successfully leverage. Additionally, participants' HEART scores changed over time in predictable ways based on the type of counseling module received.

**Conclusion:**

Overall, the tool performed as predicted and was consistent with past validation efforts of the HEART, indicating that the HEART may provide an efficient means to tailor women's counseling to address relationship-related challenges to PrEP adherence.

## Abstract (precis)

Authors examined the effectiveness of the Healthy Relationship Assessment Tool to guide counselors in supporting women to address relationship-related challenges to pre-exposure prophylaxis adherence and measured women's score changes after counseling over time.

## Teaser

**Key Findings:**

- Overall, the Healthy Relationship Assessment Tool (HEART) performed as predicted and was consistent with past validation efforts of the tool, increasing the body of evidence supporting its use as a tool to guide tailored counseling to support women's pre-exposure prophylaxis (PrEP) adherence.
- Counselors found the tool's recommendations appropriate and delivered the recommended counseling module despite being able to override recommendations based on additional knowledge of women's cases, indicating the HEART may help counselors more quickly and accurately assess a woman's relationship and tailor counseling.
- As a result of receiving the counseling modules, participants' HEART scores changed over time as expected, with a small but statistically significant effect associated with the intervention and likely with the module received.

**Key Implication:**

- Clinicians and counselors should consider implementing the HEART as an easy, effective, and efficient way to tailor counseling for women to support improved PrEP adherence.

## INTRODUCTION

Despite significant progress in the reduction of new HIV infections globally over the past 2 decades, young women in Africa remain disproportionately affected by HIV.^1^^,^^2^ The Joint United Nations Programme on HIV/AIDS reports that of the 1.7 million new HIV infections in 2019, approximately 47% globally were women; however, young women aged 15–24 years accounted for approximately 25% of all new infections in sub-Saharan Africa despite making up only 10% of the population.^2^ Previous research has suggested that inequitable and unbalanced patriarchal gender norms contribute to women's ongoing heightened risk of HIV infection, especially as the threat of intimate partner violence (IPV) in relationships limits women's abilities to adopt HIV prevention behaviors.^3^^–^^5^

An array of HIV pre-exposure prophylaxis (PrEP) products has been and continues to be developed to help reduce new HIV infections, but proper adherence to these new products remains a barrier. In several trials of daily oral PrEP among African women, low rates of adherence to and persistence on PrEP posed major barriers.^6^^–^^11^ A variety of factors contribute to adherence issues.^12^^,^^13^ Relationship challenges can be among them, but the effects of PrEP use in relationships can vary.^13^^,^^14^ Communication among couples may increase as a result of PrEP disclosure, which may, in turn, increase adherence to PrEP.^15^^–^^17^ However, difficulties with disclosure in relationships, male partner opposition in response to disclosure (even if nonviolent), and IPV in particular may, in turn, decrease PrEP adherence.^18^^,^^19^

In response to the diverse roles that male partners may play in women's decisions about and abilities to use HIV prevention products, we previously developed the counselor-administered Healthy Relationship Assessment Tool (HEART).^20^ By assessing participants' levels of partner support, control, or abuse, the HEART is used to tailor PrEP counseling and adherence support to women's relationships with their sexual partners, suggesting module-based content for counselor delivery.^20^ We piloted the HEART within a clinic-based intervention to reduce social harms and increase healthy relationship dynamics for PrEP use, the Community Health clinic model for Agency in Relationships and Safer Microbicide Adherence (CHARISMA). This pilot produced promising results for the initial validation of the tool.^20^^–^^23^ After conducting this pilot, a randomized controlled trial (RCT) to test the effectiveness of CHARISMA on a broader scale was established. During this RCT, we conducted further validation of the HEART to assess its impact in larger-scale settings. In this study, we examine (1) the extent to which counselors used the HEART to guide the delivery of intervention modules and (2) whether changes in subsequent HEART scores reflected the counseling module(s) received.

The HEART is used to tailor PrEP counseling and adherence support to women's relationships with their sexual partners, suggesting module-based content for counselor delivery.

## METHODS

### The CHARISMA RCT

Between October 2018 and October 2019, the CHARISMA RCT enrolled 407 women in Johannesburg, South Africa. Per the CHARISMA RCT eligibility requirements, participants were HIV-seronegative, nonpregnant, currently in a relationship with a male primary sexual partner, sexually active (by self-report), willing to use daily oral PrEP, and had no prior history of participation in clinical trials or longitudinal studies of HIV prevention. Participants who enrolled were offered oral PrEP for 6 months and were randomized 1:1 to additionally receive either the CHARISMA empowerment counseling intervention (treatment group, n=203) or standard of care (SOC) counseling (control group, n=204). CHARISMA counseling was composed of tailored empowerment counseling at enrollment through various counseling modules and ongoing follow-up with counseling as needed at month 1, month 3, and month 6 visits (though a longer follow-up period would be preferable, follow-up was limited to a maximum of 6 months by time and resource constraints). Tailored counseling topics included partner communication, PrEP disclosure, and responding to IPV. Also, all women received a healthy relationships module. SOC counseling consisted of supportive counseling with onward referrals to public facilities for further management.

### HEART Administration

In addition to other CHARISMA activities, trained lay counselors administered the HEART to all treatment group participants at enrollment. The original HEART contained 41 items across 5 scales: (1) Traditional Values, (2) Partner Support, (3) Partner Abuse and Control, (4) Partner Resistance to HIV Prevention, and (5) HIV Prevention Readiness. Based on subsequent psychometric analysis, we reduced the number of items in some scales.^24^ Table 1 describes the final reduced set of HEART scales used in this analysis.

**TABLE 1.** Summary of HEART Scales Used in the CHARISMA PrEP Adherence Counseling Intervention, Johannesburg, South Africa

Scale and Description	Number of Items
Traditional Values: Norms valuing masculinity (e.g., “A man should have the final say in all family matters.”)	9
Partner Support: Ways that the relationship with her partner is/is not supportive or harmonious (e.g., “My partner is as committed as I am to our relationship.”)	6
Partner Abuse and Control: Partner's psychologically or physically abusive behaviors or their outcomes (e.g., “My partner makes fun of me or humiliates me.”)	7
Partner Resistance to HIV Prevention: Partner's unwillingness to talk about or use HIV prevention (e.g., “If I asked my partner to use a condom, he would get angry.”)	5
HIV Prevention Readiness: Individual or joint readiness to use HIV prevention products (e.g., “Using PrEP with my partner will help us communicate better.”)	5

Abbreviations: CHARISMA, Community Health clinic model for Agency in Relationships and Safer Microbicide Adherence; HEART, Healthy Relationship Assessment Tool; PrEP, pre-exposure prophylaxis.

The HEART was administered electronically by a counselor via a tablet. For each scale, a score was automatically calculated for each participant by averaging her item responses. Those scores were then compared to calibrated cut-points indicating “risk zones” that were then used to recommend counseling modules. For example, a Partner Abuse and Control score in a high-risk zone would direct to an automated recommendation for the participant to receive a module addressing IPV.^24^ However, counselors could override the HEART recommendation based on additional insights they obtained about a participant's relationship dynamics during the introductory session, allowing them to either decline the module recommended by HEART or direct participants to a module not recommended by HEART. For example, explicit participant disclosure of experience of IPV would result in an automatic recommendation for the responding to IPV module (in addition to referrals to external providers for additional support as needed, similar to SOC). An indication that the participant had not disclosed ring use to her partner would result in an automatic recommendation for the PrEP disclosure module. The HEART was administered to all treatment group participants at enrollment, all returning treatment group participants at month 3, and all returning participants in both the treatment and control groups at month 6.

Final study visits for some CHARISMA participants took place January–April 2020, coinciding with the imposition of lockdown risk mitigation strategies to manage the COVID-19 epidemic in South Africa. Rather than bringing in the remaining study participants for full CHARISMA RCT exit visits, some final participants received an abbreviated set of procedures that did not include final administration of the HEART.

### Analytic Approach

In this analysis, we used the HEART scores and data from the CHARISMA RCT to validate the HEART. We compared (1) the HEART scale scores at enrollment by counseling module received, (2) the HEART scale scores over time for participants in the intervention arm, and (3) the HEART scale scores of the treatment and control groups at month 6. We hypothesized that if the HEART performed as expected, mean scale scores at enrollment should differ by counseling module recommendation. Table 2 describes how we specifically anticipated mean scores on each HEART subscale to trend for different counseling modules at enrollment. We expected that over time and regardless of counseling module, mean scores for Traditional Values, Partner Abuse and Control, and Partner Resistance would decrease, and mean scores for Partner Support and HIV Prevention Readiness would increase. Finally, we anticipated that the increases and decreases in mean scores previously described would result in statistically significant differences between the treatment group and the control group, with higher mean scores in the treatment group for Traditional Values, Partner Abuse and Control, and Partner Resistance and lower scores for Partner Support and HIV Prevention Readiness. For all hypotheses, we assessed differences in means using t-tests at the 90%, 95%, and 99% levels for statistical significance.

**TABLE 2.** Hypothesized Direction of HEART Scale Scores at Enrollment in the CHARISMA PrEP Adherence Counseling Intervention, by Counseling Module

Scale	Partner Communication	PrEP Disclosure	Responding to IPV
Traditional Values	Lower	Lower	Higher
Partner Support	Higher	Higher	Lower
Partner Abuse and Control	Lower	Lower	Higher
Partner Resistance	Higher	Lower	Higher
HIV Prevention Readiness	Higher	Higher	Lower

Abbreviations: CHARISMA, Community Health clinic model for Agency in Relationships and Safer Microbicide Adherence; HEART, Healthy Relationship Assessment Tool; IPV, intimate partner violence; PrEP, pre-exposure prophylaxis.

## RESULTS

Table 3 indicates the number of HEART administrations by study arm at each time point. A total of 73 participants from the intervention group and 67 from the control group were either lost to follow-up or received abbreviated study exit procedures by the month 6 follow-up administration.

**TABLE 3.** Number of Participants Assessed by HEART in the CHARISMA PrEP Adherence Counseling Intervention, by Study Arm and Visit Type

Visit	Control (n=204 enrolled)	Treatment (n=203 enrolled)
Enrollment	0	203
Month 3 follow-up	0	157
Month 6 follow-up	137	130

Abbreviations: CHARISMA, Community Health clinic model for Agency in Relationships and Safer Microbicide Adherence; HEART, Healthy Relationship Assessment Tool; PrEP, pre-exposure prophylaxis.

As previously described, though the HEART included automated recommendations for counseling modules based on subscale risk zones, counselors had the ability to override the HEART recommendation based on additional knowledge. Table 4 compares the number of participants recommended to receive each counseling module by the HEART against the actual counselor module decision. Overall, counselors agreed with HEART recommendations about which counseling modules were appropriate, differing in less than 5% of cases.

**TABLE 4.** Number of Participants Recommended Counseling Modules by HEART Versus Counselor Decision at Enrollment in the CHARISMA PrEP Adherence Counseling Intervention

	Counselor Decision		
	Partner Communication	PrEP Disclosure	Responding to IPV
HEART recommendation			
Partner Communication	129	0	1
PrEP Disclosure	2	50	4
Responding to IPV	0	2	15
Total	131	52	20

Abbreviations: CHARISMA, Community Health clinic model for Agency in Relationships and Safer Microbicide Adherence; HEART, Healthy Relationship Assessment Tool; IPV, intimate partner violence; PrEP, pre-exposure prophylaxis.

### How Do HEART Scale Scores Differ by Recommended Module?

We explored how women differed on their enrollment HEART scale scores based on their counselor's module recommendation. Table 5 summarizes the mean score and standard deviation of each HEART scale, disaggregated by the counseling module to which the participants were referred (Supplement Figure S1 provides score distributions).

**TABLE 5.** Mean and Standard Deviation of HEART Scale Scores in the CHARISMA PrEP Adherence Counseling Intervention, by HEART Recommendation at Enrollment With Pair-Wise Comparisons

	Module Recommended			Pair-Wise Comparison, Student's *t*		
Scale	Partner Communication (A) (n=131)	PrEP Disclosure (B) (n=52)	Responding to IPV (C) (n=20)	(A) vs. (B) (n=183)	(A) vs. (C) (n=151)	(B) vs. (C) (n=72)
Traditional Values	1.54 (0.85)	1.62 (0.87)	1.83 (0.95)	0.55	1.39	0.90
Partner Support	5.41 (0.76)	5.10 (0.92)	3.09 (1.14)	2.31^a^	11.81^b^	7.74^b^
Partner Abuse and Control	1.25 (0.43)	1.36 (0.64)	3.49 (1.15)	1.33	16.15^b^	9.93^b^
Partner Resistance	1.29 (0.59)	1.63 (0.90)	3.44 (1.26)	2.95^b^	12.57^b^	6.80^b^
HIV Prevention Readiness	5.53 (0.70)	5.17 (1.00)	4.69 (0.97)	2.68^b^	4.68^b^	1.85^c^

Abbreviations: CHARISMA, Community Health clinic model for Agency in Relationships and Safer Microbicide Adherence; HEART, Healthy Relationship Assessment Tool; IPV, intimate partner violence; PrEP, pre-exposure prophylaxis.

a Statistically significant at the 0.05 level.

b Statistically significant at the 0.01 level.

c Statistically significant at the 0.10 level.

The most significant differences in scale scores were in the Partner Support, Partner Abuse and Control, and Partner Resistance scales when comparing those who were recommended the IPV module to those who were recommended 1 of the other 2 modules, either the partner communication module or the PrEP disclosure module. As expected, women who were recommended to receive the responding to IPV module scored lower in Partner Support and higher in Partner Abuse and Control and in Partner Resistance scales.

All 3 groups scored low and not statistically different in the Traditional Values scale and scored high with statistically significant differences in the HIV Prevention Readiness scale. For the latter, participants who were recommended the partner communication module scored the highest (mean 5.53; standard deviation [SD] 0.70), followed by those who were recommended the PrEP disclosure module (mean 5.17; SD 1.00), and then by those who were recommended the responding to IPV module (mean 4.69; SD 0.97).

### How Do HEART Scale Scores Change Over Time After Receiving a Counseling Session?

In addition to understanding whether the HEART could be used to sort participants and recommend counseling modules at enrollment, we sought to understand whether and how the HEART scale scores would change after receiving the modules. The Figure shows the mean score of each HEART scale at enrollment, at month 3, and at month 6, disaggregated by the module to which participants were referred (Supplement Table S1).

**FIGURE.**
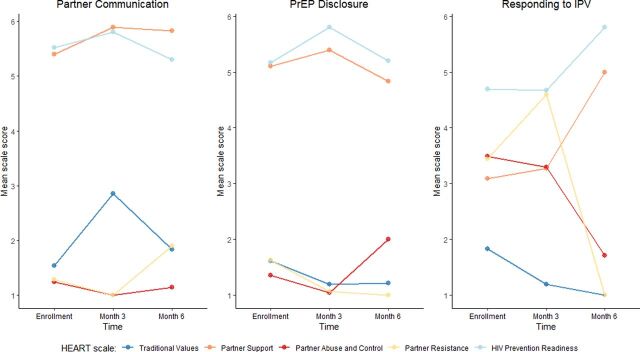
Healthy Relationship Assessment Tool Scale Score Over Time, by Module Received at Enrollment in the CHARISMA PrEP Adherence Counseling Intervention, Johannesburg, South Africa Abbreviations: CHARISMA, Community Health clinic model for Agency in Relationships and Safer Microbicide Adherence; IPV, intimate partner violence; PrEP, pre-exposure prophylaxis.

For participants who were recommended the responding to IPV module, there were substantial changes across all 5 scales, particularly in the Partner Support, the Partner Abuse and Control, and the Partner Resistance scales. Mean scores on the Partner Support scale increased from 3.09 at enrollment to 3.27 at month 3 and 5.00 at month 6; the Partner Abuse and Control scale mean scores decreased from 3.49 at enrollment to 3.29 at month 3 and 1.71 at month 6; and the Partner Resistance scale mean scores also changed from 3.44 at enrollment to 4.60 at month 3 and 1.00 at month 6. Conceptually, these changes reflect participants' perceptions that their partners had become more supportive in their relationships (from the Partner Support scale), were exhibiting less abusive and controlling behavior in their relationships (from the Partner Abuse and Control scale), and had become less resistant to the use of HIV prevention products in their relationships (from the Partner Resistance scale). Changes in the Traditional Values and in the HIV Prevention Readiness scales were smaller but also in the expected direction (i.e., decreases in gendered traditional values and increases in readiness to use HIV prevention products). In turn, mean scores in the Traditional Values scale decreased from 1.83 at enrollment to 1.19 at month 3 and 1.00 at month 6, and mean scores in the HIV Prevention Readiness scale changed from 4.69 at enrollment to 4.67 at month 3 and 5.80 at month 6.

For participants who received the partner communication or the PrEP disclosure modules, the changes across all 5 scales were largely in the expected direction but of much smaller magnitude, seemingly because for these 2 groups the scores at enrollment were already close to the lower and upper bounds of each respective scale range.

### Do HEART Scores Differ by Intervention Arm at the 6-Month Visit?

Finally, we sought to compare the HEART scores of the 2 study arms at month 6. Table 6 summarizes the mean HEART scores for each scale by intervention status (Supplement Figure S2 for score distributions). At the 6-month visit, participants assigned to the intervention arm scored higher in Partner Support and HIV Prevention Readiness (meaning they perceived their partners as more supportive in their relationships and felt more prepared to use HIV prevention products) and lower in Traditional Values, Partner Abuse and Control, and Partner Resistance (meaning they held less gendered traditional values and perceived their partners as less abusive or controlling and less resistant to using HIV prevention products in their relationships). Although the differences were small, they were all statistically significant at the 0.05 level (and except for the difference in Partner Resistance scale scores, all were statistically significant at the 0.01 level).

**TABLE 6.** Mean and Standard Deviation of HEART Scale Scores in the CHARISMA PrEP Adherence Counseling Intervention, by Intervention Arm at 6-Month Visit

HEART Scale	Control (n=137)	Treatment (n=130)	Student's *t*
Traditional Values	1.53 (0.82)	1.14 (0.29)	5.08^a^
Partner Support	5.13 (1.08)	5.64 (0.65)	−4.66^a^
Partner Abuse and Control	1.36 (0.71)	1.08 (0.22)	4.34^a^
Partner Resistance	1.36 (0.83)	1.18 (0.48)	2.12^b^
HIV Prevention Readiness	5.65 (0.64)	5.82 (0.42)	−2.45^a^

Abbreviations: CHARISMA, Community Health clinic model for Agency in Relationships and Safer Microbicide Adherence; HEART, Healthy Relationship Assessment Tool; PrEP, pre-exposure prophylaxis.

a Statistically significant at the 0.01 level.

b Statistically significant at the 0.05 level.

## DISCUSSION

In this study, we examined the effectiveness of the HEART in guiding the delivery of the CHARISMA intervention counseling modules and measured the change in HEART scale scores over time after their implementation. Overall, the tool performed as predicted within an RCT and was consistent with past validation efforts of the HEART. These results further increase the body of evidence supporting use of the HEART as a tool to guide tailored counseling to support women's PrEP adherence.

At enrollment, the counselors bore the ultimate decision about which module a participant would receive. In the small number of cases in which there were differences, the most frequent difference was to opt to include the module on responding to IPV even when not recommended. This demonstrates the importance of allowing some flexibility in the implementation of the HEART, as these cases could be informed by additional outside information or by counselor choice to select a more protective, risk-averse path for the participant in the face of any uncertainty (receiving the module when not indicated by HEART may still be beneficial and is unlikely to be harmful). However, most counselors, when considering the full scope of information known about a woman's specific case, agreed with the HEART's module recommendation. This indicates that the HEART may help counselors more quickly but still accurately assess a woman's relationship and tailor counseling.

Most counselors, when considering the full scope of information known about a woman's specific case, agreed with the HEART's module recommendation.

Because the counselor's module recommendations were largely aligned with the HEART module recommendations, these differences in the HEART scores naturally reflect the module assignment mechanism. Specifically, the Partner Support, Partner Abuse and Control, and Partner Resistance scales contributed to the module assignment mechanism. It is worth emphasizing that although counselors were given the faculty to override the HEART module recommendation, in most cases, they chose to follow the recommendation. By doing so, they implicitly also validated the scale scores (counselors were shown the HEART recommendation but not the actual scale scores). We also note the groups differed not only on the scales used to make the recommendations, as expected, but also on other scales that did not inform the recommendations.

Additionally, women referred to the partner communication module had significantly higher HEART scores in Partner Support and HIV Prevention Readiness and lower scores in Partner Abuse and Control and Partner Resistance to HIV Prevention that positioned them to successfully leverage the module to communicate with their partners. Similarly, women referred to the responding to IPV module were prime candidates to benefit from this module, according to the HEART, being the only group with significantly higher Partner Abuse and Control scores (in addition to higher scores in Partner Resistance to HIV Prevention and lower scores in Partner Support and HIV Prevention Readiness). Women recommended to receive the PrEP disclosure module were not significantly different on HEART scores than those not recommended to the module, but their scores did trend in an expected direction. These women scored higher on Partner Resistance to HIV Prevention and lower on HIV Prevention Readiness but not higher on Partner Abuse and Control, which may describe relationships in which women feel a relatively low risk of IPV but have difficulty discussing HIV prevention with their partners.

The Traditional Values scale is not currently used to recommend counseling modules, nor does it seem to identify differences in women as strongly as the other scales. However, because these items were positioned as the first to be asked in the HEART, this scale may have provided additional helpful background information for counselors when determining whether to follow the HEART recommendation. Although removal of the Traditional Values scale could shorten the tool, making it easier and quicker to implement, this might also reduce the utility of the HEART by varying either participants' openness to respond to the more personally focused scales that follow or by varying counselors' perceptions of participants' responses and, therefore, counselor trust in HEART recommendations. Further research into counselors' assessments of the HEART and its recommendations should be conducted before alterations are made.

We were also interested in how participants' HEART scores changed over time as a result of receiving the counseling modules. The results were generally consistent with expectations; we note a small but statistically significant effect associated with the intervention and likely with the counseling module received. Considering that participants were randomized to SOC versus CHARISMA, one can reasonably assume that these 2 groups were comparable at enrollment and that these differences are due to the intervention, partly because of the counseling module to which participants were assigned. Unfortunately, by not obtaining HEART scores for the control group at enrollment or month 3, we are limited to the month 6 comparison and thus unable to confirm that the 2 groups were statistically comparable at baseline. It is difficult to know if this trend would continue based on the 1-time implementation of the CHARISMA intervention. But, a systematic review of behavioral IPV interventions by Arroyo et al. found lingering significant behavioral outcomes for several interventions at 1-year follow-up,^25^ and Kenthirarajah and Walton provide a model for the ability of brief interventions to have long-term impacts.^26^ Therefore, it is plausible that effects may extend past the 6-month follow-up.

### Limitations

Despite promising results for the validity of the HEART, our study has some limitations. Development and validation efforts for the HEART thus far have been concentrated around Johannesburg, South Africa, particularly within a clinical trial research setting and implemented by trained staff. Although results in this setting remain promising, further research is needed to determine how both the HEART overall and its scales function outside of this setting in a real-world environment in South Africa and other regions. It is important to note, though, that implementation of the HEART has been led by lay counselors, not clinical staff, providing evidence that its utility has not been limited to specialist clinical staff. Relatedly, future research will also explore the ability of the HEART to be self-administered, which will also be informative about its use and function in other settings.

## CONCLUSION

Although possible methods to improve the HEART's validity and utility remain, this study demonstrates the tool's ability to identify key aspects of women's relationship dynamics to help tailor counseling to meet their needs. Such tailoring can help overcome the social and relationship barriers to oral PrEP adherence, but the process of understanding each participant's relationship profile can be time and resource intensive. Additionally, this study provides evidence for how the tool can measure the impact of counseling on these aspects of relationship dynamics. The availability of a tool such as the HEART could provide clinicians and counselors with an easy, effective, and efficient way to tailor counseling toward women's relationship dynamics and better address challenges to PrEP adherence.

## Supplementary Material

GHSP-D-22-00075-supplement.pdf

## Notes

**Peer Reviewed**

**First published online:** October 12, 2023.
