# Supplementary material for: Measuring Effects of Counseling to Increase Pre-Exposure Prophylaxis Adherence and Partner Support in South Africa Using the Healthy Relationship Assessment Tool
Source: Glob Health Sci Pract. 2023 Oct 30;11(5):e2200075. doi: 10.9745/GHSP-D-22-00075 (PMC10615234; doi:10.9745/GHSP-D-22-00075)

SUPPLEMENT

FIGURE S1. Distribution of Healthy Relationship Assessment Tool Scale Scores by Module Recommendation for CHARISMA Trial Participants at Enrollment

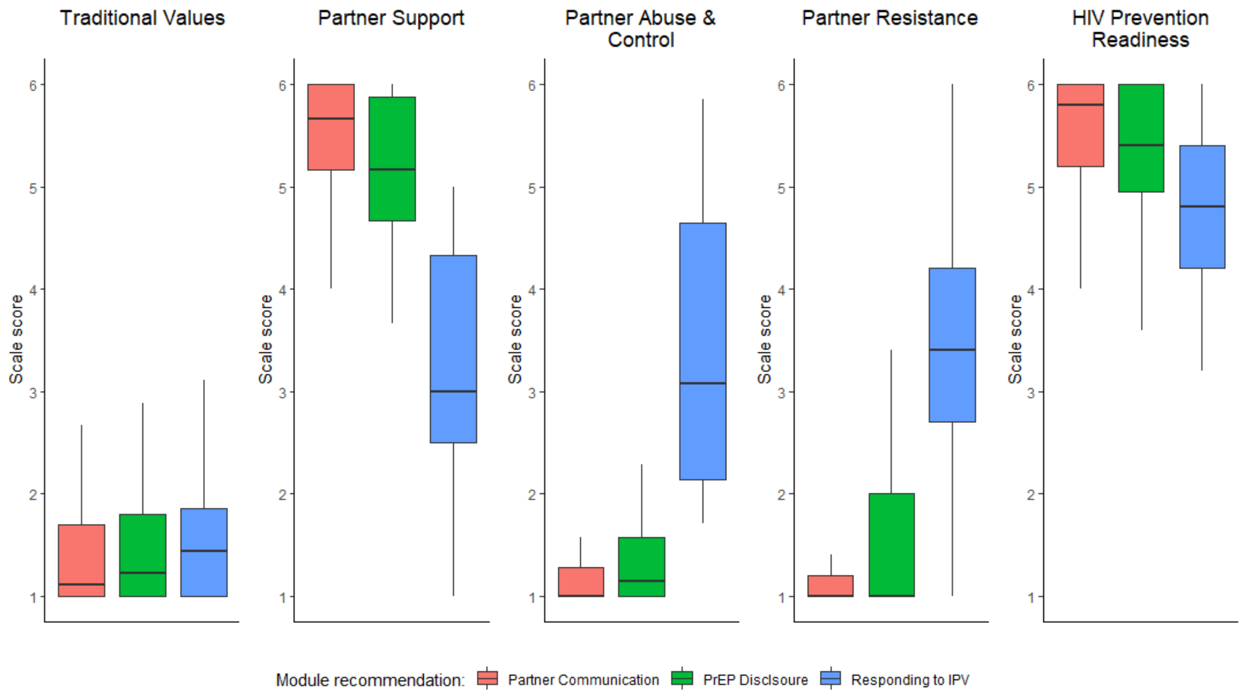

Abbreviations: CHARISMA, Community Health clinic model for Agency in Relationships and Safer Microbicide Adherence; IPV, intimate partner violence; PrEP, pre-exposure prophylaxis.

TABLE S1. Mean and Standard Deviation of Healthy Relationship Assessment Tool Scale Scores over Time by Module Received at Enrollment

**Module Received**	**Partner Communication (n=130)**			**PrEP Disclosure (n=66)**			**Responding to IPV (n=19)**		
	**Enroll-ment**	**Month 3**	**Month 6**	**Enroll-ment**	**Month 3**	**Month 6**	**Enroll-ment**	**Month 3**	**Month 6**
Traditional Values	1.54 (0.86)	2.85 (2.31)	1.83 (0.24)	1.62 (0.87)	1.19 (0.32)	1.22 (0)	1.83 (0.95)	1.19 (0.32)	1.00 (0)
Partner Support	5.40 (0.76)	5.89 (0.19)	5.83 (0.24)	5.10 (0.92)	5.39 (0.79)	4.83 (0)	3.09 (1.14)	3.27 (1.97)	5.00 (0)
Partner Abuse & Control	1.24 (0.43)	1.00 (0)	1.14 (0.20)	1.36 (0.64)	1.05 (0.08)	2.00 (0)	3.49 (1.15)	3.29 (2.23)	1.71 (0)
Partner Resistance	1.29 (0.59)	1.00 (0)	1.90 (0.71)	1.63 (0.90)	1.07 (0.12)	1.00 (0)	3.44 (1.26)	4.60 (1.22)	1.00 (0)
HIV Prevention Readiness	5.52 (0.71)	5.80 (0.20)	5.30 (0.99)	5.17 (1.00)	5.80 (0.35)	5.20 (0)	4.69 (0.97)	4.67 (0.61)	5.80 (0)

Abbreviations: IPV, intimate partner violence; PrEP, pre-exposure prophylaxis.

FIGURE S2. Distribution of Healthy Relationship Assessment Tool Scale Scores at Month 6, by Arm

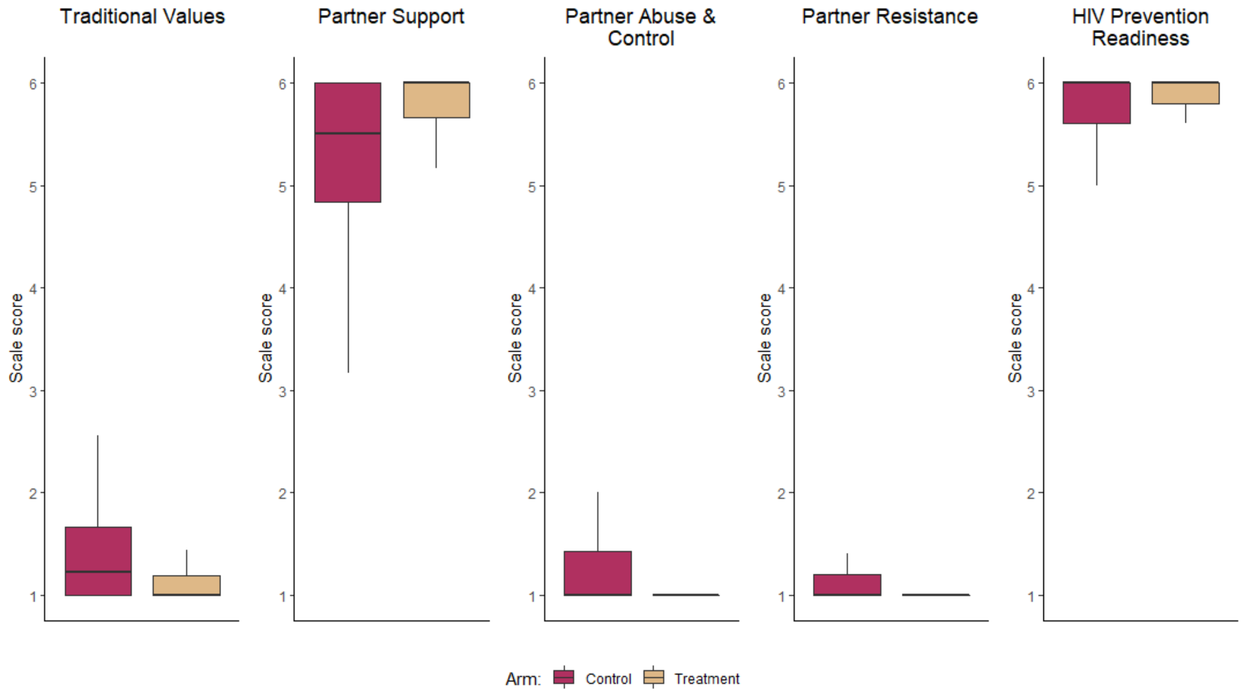

Supplement: GHSP-D-22-00075-supplement.pdf [file GHSP-D-22-00075-supplement.pdf]
